# Biotechnology Notes: A Year of Milestones

**DOI:** 10.1016/j.biotno.2024.11.004

**Published:** 2024-11-15

**Authors:** Matthew Wook Chang

**Affiliations:** a NUS Synthetic Biology for Clinical and Technological Innovation (SynCTI), National University of Singapore, Singapore; b Department of Biochemistry, Yong Loo Lin School of Medicine, National University of Singapore, Singapore; c National Centre for Engineering Biology (NCEB), Singapore

As we conclude another remarkable year in research, it's exciting to reflect on the growth of *Biotechnology Notes*. Following our Scopus indexing last year, we are thrilled to announce that *Biotechnology Notes* is now also indexed in PubMed Central—a milestone that underscores the dedication of our contributors, readers, and editorial team, while further expanding our journal's visibility and impact.

Biotechnology continues to transform approaches to some of society's greatest challenges, spanning healthcare, agriculture, biomanufacturing and environmental sustainability. The discussion about biotechnology is evolving from what biotechnology *can* achieve to how we can responsibly harness its vast potential for the benefits to society and the planet.

This year, we observed a significant increase in manuscript submissions across diverse topics. Our publications included original research, review, discussion, and communication articles, highlighting cutting-edge breakthroughs at the intersection of biology and technology. Three review articles, in particular, garnered substantial attention and became our most downloaded pieces: (i) *A comprehensive study on anaerobic digestion of organic solid waste: A review on configurations*, where Kumar et al. provided an in-depth assessment of cost-benefit considerations and challenges in anaerobic digestion of organic waste, focusing on process parameters (https://doi.org/10.1016/j.biotno.2024.02.001). (ii) *Organ-on-Chip Technology: Opportunities and Challenges*, where Srivastava et al. offered a systematic overview of the experimental advantages and advancements in Organ-on-Chip technology, specifically its applications in multiorgan systems (https://doi.org/10.1016/j.biotno.2024.01.001). (iii) *The Potential of Copper Oxide Nanoparticles in Nanomedicine: A Comprehensive Review*, where Devaraji et al. discussed the benefits of “green” copper oxide nanoparticles produced by microbes for clinical applications (https://doi.org/10.1016/j.biotno.2024.06.001).

Our publications this year covered a wide array of subjects—from foundational synthetic biology, metabolic engineering, and biomolecular engineering to an innovative software contribution. In one research article, *Identifying Chlorella vulgaris and Chlorella sorokiniana as sustainable organisms to bioconvert glucosamine into valuable biomass*, Elhalis et al. discovered that pretreatment of glucosamine with amyloglucosidase and α-amylase can positively impact microalgae biomass recovery, an essential factor in bioproduction (https://doi.org/10.1016/j.biotno.2024.01.003). In another research article, *Understanding virus retention mechanisms on protein A chromatography based on different wash buffers – Evaluating the possibility of a generic wash buffer toolbox to improve virus clearance capacity*, Krause et al. provided a better understanding of the selection of various wash buffer formulations for clearing different types of viruses during monoclonal antibody production, which could inform the design of cost-effective manufacturing processes (https://doi.org/10.1016/j.biotno.2024.03.001).

With advancements in new technologies, such as artificial intelligence, cell-free systems are increasingly being applied in both fundamental and applied research. In a notable perspective article, fourteen experts from around the world shared insights into the future of cell-free synthetic biology, a rapidly evolving field that offers new and powerful approaches to understanding natural living systems (https://doi.org/10.1016/j.biotno.2024.11.001). Collectively, the authors presented an extensive view of the challenges and opportunities through which cell-free systems can transform human health, biotechnology, and the environment.

Thank you to our authors, reviewers, and readers for helping *Biotechnology Notes* grow into an invaluable resource for the scientific community. I look forward to reaching our next milestone together.

